# Chronic COVID-19 infection in an immunosuppressed patient shows changes in lineage over time: a case report

**DOI:** 10.1186/s12985-023-02278-7

**Published:** 2024-01-04

**Authors:** Sheridan J. C. Baker, Landry E. Nfonsam, Daniela Leto, Candy Rutherford, Marek Smieja, Andrew G. McArthur

**Affiliations:** 1 David Braley Centre for Antibiotic Discovery, McMaster University, Hamilton, ON Canada; 2 Michael G. DeGroote Institute for Infectious Disease Research, McMaster University, Hamilton, ON Canada; 3 Department of Biochemistry and Biomedical Sciences, McMaster University, Hamilton, ON Canada; 4 Hamilton Regional Laboratory Medicine Program, St Joseph’s Healthcare, Hamilton, ON Canada; 5 Department of Pathology and Molecular Medicine, McMaster University, Hamilton, ON Canada; 6 Hamilton Regional Laboratory Medicine Program, Hamilton Health Sciences, Hamilton, ON Canada

**Keywords:** COVID-19, Hodgkin’s lymphoma, Cancer, Immunocompromised, COVID-19 reinfection, Chronic COVID-19

## Abstract

**Background:**

The COVID-19 pandemic, caused by the Severe Acute Respiratory Syndrome Coronavirus 2 virus, emerged in late 2019 and spready globally. Many effects of infection with this pathogen are still unknown, with both chronic and repeated COVID-19 infection producing novel pathologies.

**Case presentation:**

An immunocompromised patient presented with chronic COVID-19 infection. The patient had history of Hodgkin’s lymphoma, treated with chemotherapy and stem cell transplant. During the course of their treatment, eleven respiratory samples from the patient were analyzed by whole-genome sequencing followed by lineage identification. Whole-genome sequencing of the virus present in the patient over time revealed that the patient at various timepoints harboured three different lineages of the virus. The patient was initially infected with the B.1.1.176 lineage before coinfection with BA.1. When the patient was coinfected with both B.1.1.176 and BA.1, the viral populations were found in approximately equal proportions within the patient based on sequencing read abundance. Upon further sampling, the lineage present within the patient during the final two timepoints was found to be BA.2.9. The patient eventually developed respiratory failure and died.

**Conclusions:**

This case study shows an example of the changes that can happen within an immunocompromised patient who is infected with COVID-19 multiple times. Furthermore, this case demonstrates how simultaneous coinfection with two lineages of COVID-19 can lead to unclear lineage assignment by standard methods, which are resolved by further investigation. When analyzing chronic COVID-19 infection and reinfection cases, care must be taken to properly identify the lineages of the virus present.

## Key points

- A patient repeatedly tested positive for COVID-19 over 16 months.
- Infection progressed from one lineage to coinfection with a second lineage, before clearance of coinfection and reinfection with a third, different lineage.
- Coinfection was difficult to identify through genomic methods.

## Background

Coronavirus disease 2019 (COVID-19) is caused by Severe Acute Respiratory Syndrome Coronavirus 2 (SARS-CoV-2). As of October 22, 2023, over 771 million cases have been reported worldwide with over 6.9 million deaths as a result of COVID-19 [1]. SARS-CoV-2 primarily enters host cells by binding of its spike (S) protein to human cell-surface angiotensin-converting enzyme 2 (ACE2) receptors [2, 3]. SARS-CoV-2 is a positive-sense, single-stranded RNA virus, with a genome 29–30 kB in size, organized as methyl-capped-5″UTR-ORF1a/b-S-ORF3-E-M-ORF6-ORF7a/b-ORF8-N/ORF9b-ORF9c-ORF10-3’UTR-poly-A-tail [4–6]. The S, E, M, and N genes encode key structural proteins found in the mature virion—the Spike, Envelope, Membrane, and Nucleocapsid structures respectively [7]. COVID-19 primarily affects the respiratory tract, and manifests as an acute upper and/or lower respiratory syndrome that can vary in severity [8]. The disease can result in asymptomatic viral shedding, or symptomatic disease associated with fever, cough, fatigue, myalgia, arthralgia, rhinorrhea, sore throat, and conjunctivitis [8–10]. However, the disease can also progress to more severe outcomes, including persistent fever, hemoptysis, hypoxia, chest discomfort and/or pain, respiratory failure, and multiorgan failure [9, 10]. Impairment of smell and/or taste is also a common symptom of COVID-19 [11]. Typical, non-chronic, mild and moderate cases of COVID-19 are usually associated with improvement of symptoms about 10 days after onset of symptoms, though in rare cases the infection for persist for a number of weeks, known as chronic or long COVID-19 when symptoms last longer than 3 weeks [12, 13]. While the body of work surrounding comorbidities for COVID-19 infection remains large, relatively less information is available regarding potential comorbidities and risk factors for chronic COVID-19 infection or COVID-19 reinfection (a new COVID-19 infection unrelated to the previous infection) [14, 15], both of which were seen in this case. Changes in lineage (when a patient initially is found to be infected with a certain lineage, and a second, later test identifies infection by a new, distinct lineage) is often indicative of reinfection rather than within-host evolution [15, 16]. There remains a limited number of reports detailing cases of chronic COVID-19 infection and/or repeated infection. We present a chronic infection followed by reinfection, over a 16-month period, in a severely immunocompromised patient.

## Case presentation

### Initial diagnosis and treatment

A male, in his early fifties, and heavily immunosuppressed with history of Hodgkin’s lymphoma (HL) was initially treated with ABVD (doxorubicin, bleomycin, vinblastine, and dacarbazine) chemotherapy, and later, GDP (gemcitabine, dexamethasone, and cisplatin) chemotherapy, followed by autologous stem cell transplant (SCT) for relapsed HL one-year post-completion of initial chemotherapy. The patient was maintained on the CD30 antibody–drug conjugate Brentuximab until he was noted to again have HL disease progression, for which he underwent an allogeneic SCT 1.5 years post-autologous transplant. His post-transplant course was complicated by Epstein–Barr Virus (EBV) reactivation and associated post-transplant lymphoproliferative disorder (PTLD), requiring repeated courses of rituximab (twelve doses overall) and graft versus host disease (GVHD) of the skin, gut, and possibly lung, requiring multiple doses of prednisone.

The first episode of COVID-19 infection was one-month post-allogeneic SCT, prior to the diagnosis of PTLD. There were no other microbiological findings in the patient’s lungs. Shortly thereafter, the patient required rituximab for EBV reactivation, followed by recurrent episodes of EBV reactivation and CT-confirmed PTLD, leading to further courses of rituximab. Initial presenting symptoms of COVID-19 were mild upper respiratory tract infection (URTI) symptoms, and Bamlanivimab was received. However, four months after the initial infection, the patient was admitted to hospital with progressive cough and shortness of breath. Upon admission, the patient again tested positive for COVID-19. Treatment included Remdesivir, dexamethasone, and Bamlanivimab with good response. Six months later, the patient developed a progressive chronic cough and was eventually hospitalized (fourteen months after the initial COVID-19 infection) with shortness of breath and new diffuse bilateral lung consolidations. This admission, treatment included sotrovimab along with another course of remdesivir and dexamethasone. Despite initial improvement in respiratory status, the patient developed worsening renal dysfunction and shortness of breath along with progressive lung infiltrates, leading to respiratory failure and ultimately death. Pulmonary issues were multifactorial, including chronic COVID-19 infection, possible lung GVHD, and cardio-renal syndrome. The timeline of COVID-19 lineages, disease symptoms, and treatments received is summarized in Table 1.

**Table 1:** Approximate times of sampling for various COVID-19 lineages, as well as symptoms and disease status and treatment at these timepoints

Approximate time	COVID lineage	Symptoms/disease	Treatment
2019	None	Hodgkin’s Lymphoma	ABVD chemotherapy followed by GDP chemotherapy and autologous stem cell transplant
February 2021	None	Hodgkin’s Lymphoma disease progression, Graft versus host disease	Allogenic stem cell transplant, prednisome
March 2021	B.1.1.176	Mild upper respiratory tract infection, Epstein–Barr viral reactivation	Bamlanivimab
April 2021	B.1.1.176	Post-transplant lymphoproliferative disorder	Rituximab
June 2021	B.1.1.176		
June 2021	B.1.1.176		
July 2021	B.1.1.176	Progressive cough and shortness of breath	Remdesivir, dexamethasone, Bamlanivimab
July 2021	B.1.1.176		
July 2021	B.1.1.176		
August 2021	B.1.1.176		
January 2022	Not determined	Hospitalized with shortness of breath and bilateral lung consolidations	Sotrovimab, remdesivir, dexamethasone
May 2022	B.1.1.176/BA.1		
June 2022	BA.2.9		
June 2022	BA.2.9		
June 2022		Death	

## Genetic profiling

From March 2021 to June 2022, eleven samples from the patient were amplified for SARS-CoV-2 using the ARTIC V3 or ARTIC V4 protocol as outlined in Nasir et al. 2020 [17] and sequenced using an Illumina NextSeq platform. After sequencing, FASTQ files were analyzed via FastQC [18], barcode and adaptor sequences were removed using Trimmomatic [19], and SPAdes [20] was used to assemble genomes. The resulting genomes were analyzed using the SARS-CoV-2 Illumina GeNome Assembly Line (SIGNAL) pipeline (https://github.com/jaleezyy/covid-19-signal). After lineage assignment by Phylogenetic Assignment of Named Global Outbreak Lineages (PANGOLIN; github.com/cov-lineages/pangolin) within the SIGNAL workflow, mutation profiles and minor variants within samples were determined using breseq [21]. The most prevalent lineages in Ontario at the timepoints the patient was sampled were determined using VirusSeq Public Health of Ontario data (virusseq-dataportal.ca/explorer). Canonical sequences for the Alpha, Delta, and Omicron variants of SARS-CoV-2 and the most prevalent lineages at the time of patient samplings were downloaded from the NCBI Virus SARS-CoV-2 Data Hub (https://www.ncbi.nlm.nih.gov/labs/virus/vssi). Using these sequences, a maximum-likelihood phylogenetic tree was constructed by first carrying out single-nucleotide polymorphism (SNP) analysis using Parsnp [22] followed by maximum-likelihood phylogenetic tree construction using the RAxML-HPC BlackBox platform with the GTRGAMMA + I substitution model and automatic bootstrapping [23].

**Fig. 1:**
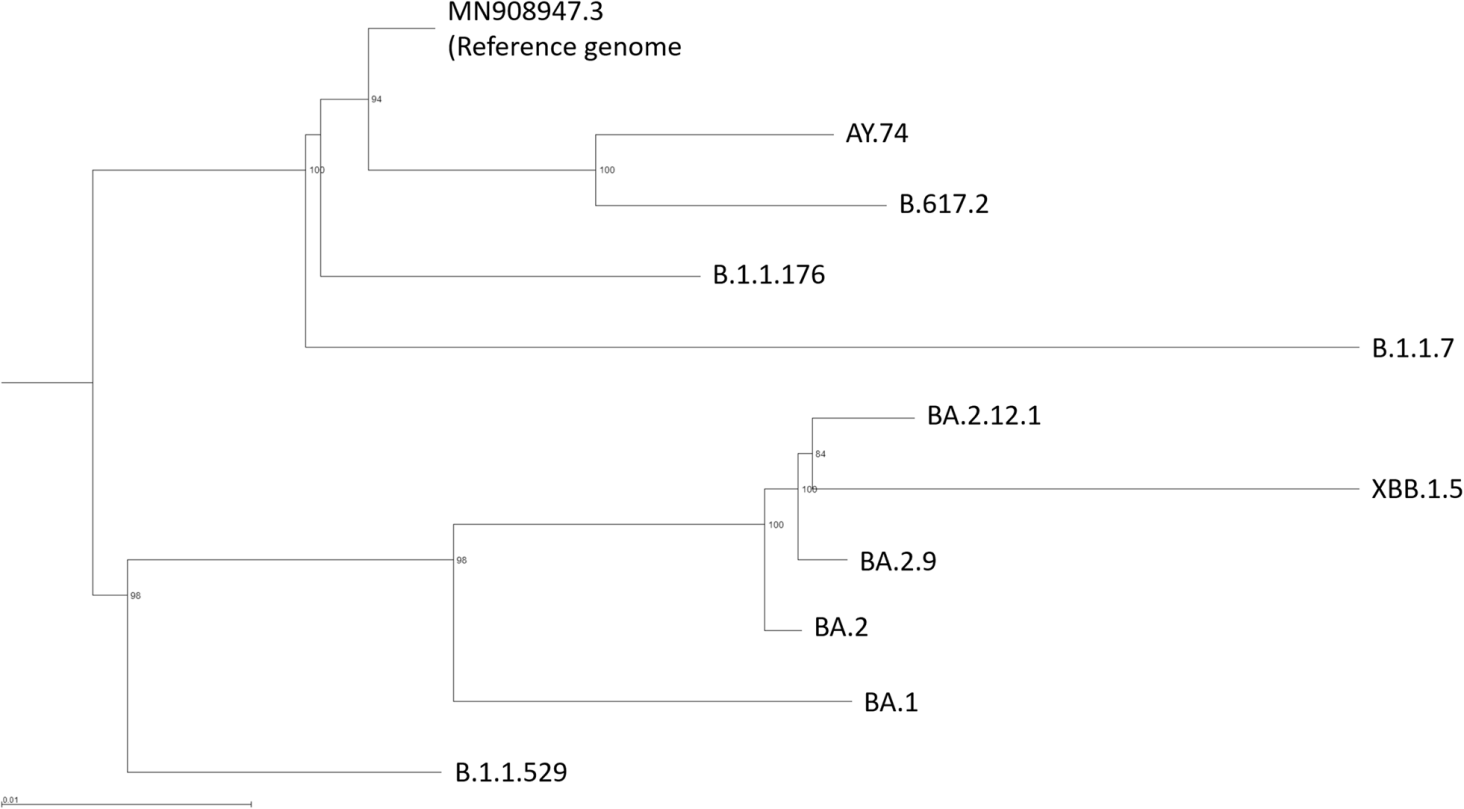
Maximum-likelihood phylogenetic tree showing SARS-CoV-2 reference sequence (MN908947.3), an Alpha lineage (B.1.1.7), a Delta lineage (B.1.617.2) and two Omicron lineages (B.1.1.529 and XBB.1.5), as well as the most prevalent lineages in Ontario at the times the patient was sampled (B.1.1.7, AY.74, BA.2, and BA.2.12.1). Bootstrap values are shown at the nodes

Lineages of patient samples, approximate dates of sampling and prevalence rates for patient lineages and most common lineages in Ontario at the time of patient samplings are shown in Table 2, and a phylogenetic tree containing canonical Alpha, Delta, and Omicron samples, the patient samples, and the most prevalent circulating strains over time in Ontario is shown in Fig. 1. Of the first eight sequenced samples, all characteristic mutations of B.1.1.176 were present with the exception of four mutations that were consistently missing in all samples. These missing mutations were L3674 in ORF1a, R203K and G204N in N, and S84LO in ORF8. There were also 10 mutations present in all eight samples that were not characteristic of B.1.1.176. These were C→T at position 241 of the genome in an intergenic region; a 3 bp deletion in ORF1; L642F, P1950L, K2029N, and N2603S in ORF1ab; C→T in the intergenic region between S and ORF3; L95F in ORF3a; a 3 bp change to AAC in N; and G→T in the intergenic region after ORF10. There were also five mutations present in the first four or five samples that were not present in samples six through eight. Present in the first four samples were S6096G in ORF1ab, T307I in S, and N269T in N; present in the first five samples were E484A and Y1155H in S. These mutations decreased in prevalence across time before not being identified in samples five or six. There were also five mutations that were gained across time, not being present in sample 7 and present in nearly 100% of reads in sample 8: a 15 bp deletion in ORF1ab, A5376V in ORF1ab, F490L and S494P in S, and T271I in N.

**Table 2:** Patient lineages across time and percentages of total lineages in Ontario made up by the lineage found in the patient

Month	patient lineage	Percentage of Ontario cases (%)	Most prevalent lineage	Percentage of Ontario cases (%)
March 2021	B.1.1.176	0.32	B.1.1.7	41.58
April 2021	B.1.1.176	0.05	B.1.1.7	36.59
June 2021	B.1.1.176	0	B.1.1.7	42.54
July 2021	B.1.1.176	0	AY.74	64.10
August 2021	B.1.1.176	0	AY.74	38.29
May 2022	B.1.1.176/BA.1	0/0	BA.2	47.17
June 2022	BA.2.9	1.16	BA.2.12.1	33.74

Also shown is the most prevalent lineage in Ontario at the same time and percentage of cases made up by the most prevalent lineage. Provincial data is from the VirusSeq data portal [55]

The next sample in the series (sample 9) was initially not assigned a lineage; however, breseq analysis revealed an infection that was a mix of B.1.1.176 and BA.1 at proportions of approximately 50% each. Mutations that were present in samples 1–8 but lost in sample 9 are shown in Table 3, while mutations that were first present in sample 9 are shown in Table 4. There were four nonsynonymous mutations present in 100% of reads in samples 1–9: C→T in the intergenic region before ORF1ab (a mutation characteristic of neither lineage), a 6 bp deletion in ORF1ab (characteristic of both B.1.1.176 and BA.1), P4715L in ORF1ab (characteristic of both B.1.1.176 and BA.1), and D614G in S (characteristic of only B.1.1.176). There were 13 nonsynonymous mutations present in samples 1–8 that were present in less than 40% of reads in sample 9 (referred to as lost mutations) and 43 nonsynonymous mutations that were present in greater than 40% of reads in sample 9 after not being present in the first 8 samples (referred to as gained mutations). These are shown in Tables 1 and 2 respectively. Of note for the gained mutations is the mutation S371F in S, which is not characteristic of either B.1.1.176 or BA.1, however the mutation S371L is characteristic of BA.1. There were also 11 nonsynonymous mutations that were not present in sample 1, appeared in some of samples 2–8, and were not present in sample 9, or were present in the intermediate samples, lost, and then reappeared in sample 9. These are shown in Table 5 and are referred to as fleeting mutations.

**Table 3:** Mutations lost in sample 9

Position	Mutation	Lineage characteristic of	Sample 1 (%)	Sample 2 (%)	Sample 3 (%)	Sample 4 (%)	Sample 5 (%)	Sample 6 (%)	Sample 7 (%)	Sample 8 (%)	Sample 9 (%)	Annotation	Gene
315	Δ3 bp	Neither	100	100	100	100	100	100	100	100	14.10	coding (50–52/13203 nt)	*orf1ab* →
1,321	A → C	B.1.1.176	58.60	92.70	86.30	91.70	77.50	23.20	100	100	23.00	E352D (GAA → GAC)	*orf1ab* →
2,189	C → T	Neither	100	100	100	100	100	100	100	100		L642F (CTT → TTT)	*orf1ab* →
6,114	C → T	Both	100	100	100	100	100	100	100	100	27.50	P1950L (CCT → CTT)	*orf1ab* →
6,352	G → T	Neither	100	100	100	100	100	100	100	100	36.10	K2029N (AAG → AAT)	*orf1ab* →
8,208	C → T	Neither	100	100	100	100	100	100	100	100	37.20	T2648I (ACT → ATT)	*orf1ab* →
14,767	G → T	B.1.1.176	100	100	100	100	100	100	100	100	30.30	A4835S (GCT → TCT)	*orf1ab* →
21,621	C → T	B.1.1.176	100	100	100	100	100	100	100	100	37.60	T20I (ACC → ATC) ‡	*S* →
22,632	G → A	B.1.1.176	100	100	100	100	100	100	100	100	33.30	R357K (AGA → AAA)	*S* →
25,386	C → T	Neither	100	100	100	100	100	100	100	100	30.70	intergenic (+ 2/-7)	*S* → / → *ORF3a*
25,947	G → T	B.1.1.176	100	100	100	100	100	100	100	100	16.60	Q185H (CAG → CAT)	*ORF3a* →
28,361	G → A	B.1.1.176	100	100	100	100	100	100	100	100	33.40	G30R (GGA → AGA)	*N* →
28,905	C → T	B.1.1.176	100	100	100	100	100	100	100	100	29.40	A211V (GCT → GTT)	*N* →

Percentages reflet the proportion of sequencing reads with the listed mutation. "Neither" indicates that the mutation is not characteristic of B.1.1.176 or BA.1. "Both" indicates that the mutation is characteristic of both B.1.1.176 and BA.1, while a blank cell indicates that the listed mutation was present in 0% of reads

**Table 4:** Mutations gained in sample 9

Position	Mutation	Lineage characteristic of	Sample9 (%)	Annotation	Gene
2,832	A → G	BA.1	58.50	K856R (AAG → AGG)	*orf1ab* →
6,513	Δ3 bp	Neither	66.00	coding (6248–6250/13203 nt)	*orf1ab* →
8,393	G → A	BA.1	78.60	A2710T (GCT → ACT)	*orf1ab* →
10,029	C → T	BA.1	57.90	T3255I (ACC → ATC)	*orf1ab* →
10,449	C → A	BA.1	63.50	P3395H (CCC → CAC)	*orf1ab* →
11,537	A → G	BA.1	54.50	I3758V (ATT → GTT)	*orf1ab* →
18,163	A → G	BA.1	51.60	I5967V (ATA → GTA)	*orf1ab* →
21,762	C → T	BA.1	58.20	A67V (GCT → GTT)	*S* →
21,766	Δ6 bp	BA.1	75.50	coding (204–209/3822 nt)	*S* →
21,846	C → T	BA.1	59.90	T95I (ACT → ATT)	*S* →
21,987	Δ9 bp	BA.1	65.60	coding (425–433/3822 nt)	*S* →
22,194	11 bp → 17 bp	BA.1	58.60	coding (632–642/3822 nt)	*S* →
22,578	G → A	BA.1	64.50	G339D (GGT → GAT)	*S* →
22,599	G → A	Neither	62.20	R346K (AGA → AAA)	*S* →
22,673	T → C	BA.1	58.00	S371P (TCC → CCC) ‡	*S* →
22,674	C → T	Neither, but S371L is hallmark of BA.1	62.20	S371F (TCC → TTC) ‡	*S* →
22,679	T → C	BA.1	62.20	S373P (TCA → CCA)	*S* →
22,686	C → T	BA.1	61.00	S375F (TCC → TTC)	*S* →
22,992	G → A	BA.1	88.90	S477N (AGC → AAC)	*S* →
22,995	C → A	BA.1	49.10	T478K (ACA → AAA)	*S* →
23,040	A → G	BA.1	53.10	Q493R (CAA → CGA)	*S* →
23,048	G → A	BA.1	48.10	G496S (GGT → AGT)	*S* →
23,055	A → G	BA.1	51.30	Q498R (CAA → CGA)	*S* →
23,063	A → T	BA.1	50.70	N501Y (AAT → TAT)	*S* →
23,075	T → C	BA.1	51.90	Y505H (TAC → CAC)	*S* →
23,202	C → A	BA.1	56.30	T547K (ACA → AAA)	*S* →
23,525	C → T	BA.1	100	H655Y (CAT → TAT)	*S* →
23,599	T → G	BA.1	49.30	N679K (AAT → AAG)	*S* →
23,604	C → A	BA.1	64.20	P681H (CCT → CAT)	*S* →
23,854	C → A	BA.1	46.20	N764K (AAC → AAA)	*S* →
23,948	G → T	BA.1	44.30	D796Y (GAT → TAT)	*S* →
24,130	C → A	BA.1	83.70	N856K (AAC → AAA)	*S* →
24,424	A → T	BA.1	61.70	Q954H (CAA → CAT)	*S* →
24,469	T → A	BA.1	62.20	N969K (AAT → AAA)	*S* →
24,503	C → T	BA.1	62.40	L981F (CTT → TTT)	*S* →
26,270	C → T	BA.1	62.80	T9I (ACA → ATA)	*E* →
26,530	A → G	BA.1	82.30	D3G (GAT → GGT)	*M* →
26,577	C → G	BA.1	54.10	Q19E (CAA → GAA)	*M* →
26,709	G → A	BA.1	59.20	A63T (GCT → ACT)	*M* →
27,807	C → T	Neither	64.90	intergenic (+ 48/-87)	*ORF7a* → / → *ORF8*
28,271	A → T	Neither	67.50	intergenic (+ 12/-3)	*ORF8* → / → *N*
28,311	C → T	BA.1	65.30	P13L (CCC → CTC)	*N* →
28,363	Δ9 bp	Neither	65.10	coding (90–98/1260 nt)	*N* →
2,832	A → G	BA.1	58.50	K856R (AAG → AGG)	*orf1ab* →

Percentages reflect the proportion of sequencing reads with the listed mutation. "Neither" indicates that the mutation is not characteristic of B.1.1.176 or BA.1

**Table 5:** Fleeting mutations

Position	Mutation	Lineage characteristic of	Sample 1 (%)	Sample 2 (%)	Sample 3 (%)	Sample 4 (%)	Sample 5 (%)	Sample 6 (%)	Sample 7 (%)	Sample 8 (%)	Sample 9 (%)	Annotation	Gene
8,477	T → A	Neither				52.60	24.00					L2738I (TTA → ATA)	*orf1ab* →
11,276	Δ20 bp	Neither				78.50						coding (11,011–11030/13203 nt)	*orf1ab* →
11,283	Δ20 bp	Neither				50.30						coding (11,018–11037/13203 nt)	*orf1ab* →
11,283	Δ15 bp	Neither								94.50		coding (11,018–11032/13203 nt)	*orf1ab* →
11,287	Δ9 bp	Neither				100	100				100	coding (11,022–11030/13203 nt)	*orf1ab* →
16,474	A → G	Neither		23.80	55.90	12.20	21.70				27.60	S5404G (AGT → GGT)	*orf1ab* →
23,013	A → C	BA.1	100	84.20	88.40	77.10	89.70				94.00	E484A (GAA → GCA)	*S* →
23,042	T → C	Neither	14.40	9.80	18.90	8.80	100		100	5.30		S494P (TCA → CCA)	*S* →
25,792	C → T	Neither		64.80	84.10	23.60	8.00	59.50			18.90	R134C (CGT → TGT)	*ORF3a* →
27,509	C → T	Neither		26.60	50.40	18.60					37.40	T39I (ACA → ATA)	*ORF7a* →
29,085	C → T	Neither		10.90		20.60	11.90	85.70		100	5.20	T271I (ACA → ATA)	*N* →

Percentages reflect the proportion of sequencing reads with the listed mutation. "Neither" indicates that the mutation is not characteristic of B.1.1.176 or BA.1, while a blank cell indicates that the listed mutation was present in 0% of reads

While sample 9 appeared to be a mixed infection of B.1.1.176 and BA.1, samples 10 and 11 were both assigned the lineage of BA.2.9, with 50 of 56 nonsynonymous mutations characteristic of BA.2.9. The mutations not characteristic of BA.2.9 (Table 6) were A5620S in ORF1ab, a 3 bp change to CTC in ORF6, C→T in the intergenic region between ORF7a and ORF8, A→T in the intergenic region between ORF8 and N, a 3 bp change to AAC in N, and a 26 bp deletion in the intergenic region after ORF10. Yet, samples 9 and 10 were missing 5 characteristic mutations of BA.2.9: L24S in S, D61L in ORF6, S84L in ORF8, and R203K and G204R in N.

**Table 6:** Mutations found in samples 10 and 11 that are not characteristic of BA.2.9

Gene	Mutation
ORF1ab	A5620S
ORF6	3 bp →CTC
Intergenic between ORF7a and ORF8	C→T
Intergenic between ORF8 and N	A→T
N	3 bp →AAC
Intergenic after ORF10	26 bp deletion

There were four mutations present in all 11 samples that were identified in 100% of reads: P4715L in ORF1ab and D614G in S (both of which are found in all three of B.1.1.176, BA.1, and BA.2.9), C→T in the intergenic region before ORF1a, and a 3 bp change to AAC in N. The latter two mutations are not characteristic of any of B.1.1.176, BA.1, or BA.2.9.

## Discussion

Several previous studies have identified cancer, and specifically hematologic cancers, as a comorbidity that worsens the health outcomes of those infected with respiratory infections such as COVID-19 [24–26]. One case report by Yonal-Hindilerden et al. reported on a patient with Hodgkin’s lymphoma who additionally contracted COVID-19. This patient experienced severe respiratory disease, eventually succumbing to COVID-19 10 days after hospital admission [27]. A second study reported on a patient with Hodgkin’s lymphoma showed reinfection with COVID-19 34 days after clearance of their initial infection [28]. However, neither of these two studies performed whole genome sequencing to assess any lineage changes in the viral infection present in the patients.

One 2021 study found that 0.47% of COVID-19 patients were incidences of reinfection [29]. Of these patients that were reinfected, the majority (67%) were reinfected with a different genomic variant than their original infection [29], as was seen in the present case. The likelihood that the three observed lineages represent within-host evolution is extremely low as B.1.1.176 and BA.1 are evolutionary very distant, with BA.1 and BA.2.9 also being genetically distinct. Another study reported on a chronic SARS-CoV-2 infection lasting over 400 days, with a SARS-CoV-2 mutation rate approximately two-fold higher than the global SARS-CoV-2 evolutionary rate [30]. This study also reported the presence over time of three genetically distinct genotypes within the patient, representing three different viral populations originating from different physical locations within the patient that continually migrated into the nasopharynx [30]. This is contrasted with the present study, where mixed infection only appears in sample 9, where the lineages B.1.1.176 and BA.1 appeared to both be present in the nasopharynx. By the next sample in the series, the lineage BA.2.9 appeared to make up 100% of the viral particles sequenced from the nasopharynx.

Immunocompromised patients are at a higher risk of chronic infection, likely due to their B-cell depleted state [31–33], as B-cells play a large role in protective immunity against SARS-CoV-19 [34]. The changes in viral lineage over time may have been associated with a poor health outcome in this patient, as Omicron lineages (BA.1 and BA.2) are associated with higher infectivity and immune escape compared to B.1.1.176 (an Alpha lineage) [35–38]. Additionally, Omicron lineages are associated with a higher risk of reinfection [39]. Further complicating the progression from an Alpha COVID-19 infection to two latter Omicron infections was the immunocompromised status of the patient, which has been found to be associated with severe clinical outcomes in COVID-19 infections [40]. Cancer patients are classified as a population at high-risk for poor health outcomes in COVID-19 infections due to their immunosuppressive state, with COVID-19 symptoms often more severe in this population [41–43]. Again compounding this risk factor in this patient was the fact that they received SCT, another factor which increases the risk of COVID-19 morbidity and mortality [44, 45]. Various studies have shown that due to their immunocompromised state, these patients are at risk for reinfection [46, 47], with one study finding that an immunocompromised patient had a higher viral load than a comparable healthy individual [48].

## Conclusion

It has been well characterized that immunocompromised individuals are at a higher risk of developing a chronic SARS-CoV-2 infection [49–54]. The present study reports a patient that was initially infected with B.1.1.176, which persisted for fifteen months, before subsequent additional infection with BA.1. When resampled one month later, the patient had apparently cleared the B.1.1.176 and BA.1 infections and had been reinfected again, this time with BA.2.9. This case thus represents incidences of chronic infection, mixed infection, as well as independent COVID-19 reinfection. The results of this case study highlight the need to closely monitor those patients that are both infected with COVID-19 and are in an immunocompromised state. To our knowledge, this case study represents one of the longest chronic COVID infections combined with reinfection in an immunocompromised patient.

## Abbreviations

COVID-19: Coronavirus disease 2019
SARS-CoV-2: Severe acute respiratory syndrome coronavirus 2
ACE2: Angiotensin converting enzyme 2
S gene: Spike gene
E gene: Envelope gene
M gene: Membrane gene
N gene: Nucleocapsid gene
HL: Hodgkin’s lymphoma
ABVD: Doxorubicin, bleomycin, vinblastine, and dacarbazine
GDP: Gemcitabine, dexamethasone, and cisplatin
SCT: Stem cell transplant
SNP: Single-nucleotide polymorphism
EBV: Epstein–Barr virus
PTLD: Post-transplant lymphoproliferative disorder
GVHD: Graft versus host disease
SIGNAL: SARS-CoV-2 Illumina GeNome Assembly Line
PANGOLIN: Phylogenetic assignment of named global outbreak lineages
